# The Pathogenesis of Eosinophilic Asthma: A Positive Feedback Mechanism That Promotes Th2 Immune Response *via* Filaggrin Deficiency

**DOI:** 10.3389/fimmu.2021.672312

**Published:** 2021-08-13

**Authors:** Wei Gao, Jiuyu Gong, Mi Mu, Yujin Zhu, Wenjuan Wang, Wen Chen, Guojing Han, Hong Hu, Pengtao Bao

**Affiliations:** ^1^Respiratory and Critical Care Unit, 1st Medical Center of Chinese Chinese People’s Liberation Army (PLA) General Hospital, Beijing, China; ^2^Department of Internal Medicine, Hubei Province Corps Hospital of The Chinese Armed Police Force (CAPF), Wuhan, China; ^3^Pulmonary and Critical Care Medicine College of Chinese PLA General Hospital, 8th Medical Center of Chinese PLA General Hospital, Beijing, China; ^4^Department of Internal Medicine, Tianjin Municipal Corps Hospital of CAPF, Tianjin, China; ^5^Department of Dermatology, 1st Medical Center of Chinese PLA General Hospital, Beijing, China; ^6^Department of Pathology, 8th Medical Center of Chinese PLA General Hospital, Beijing, China

**Keywords:** eosinophilic asthma, single nucleotide polymorphisms (SNP), FLG, Th2 immune response, IL-33, TSLP

## Abstract

Eosinophilic asthma (EA) is a common subtype of asthma and often progresses to severe disease. In order to understand its pathogenesis, targeted next-generation gene sequencing was performed on 77 Chinese EA patients and 431 Chinese healthy controls to obtain differential genomic variations. Among the 41 Single Nucleotide Polymorphisms (SNPs) screened for mutation sites in more than 3 patients, filaggrin gene *FLG* rs192116923 T>G and *FLG* rs75235053 C>G were newly found to be associated with EA patients with atopic dermatitis (AD) (P <0.001) and severe EA (P=0.032), respectively. Filaggrin has been shown to be mainly expressed in epithelial cells and plays an important role in formation of an effective skin barrier. Bioinformatic analysis indicated *FLG* rs192116923 T>G may increase the binding of Smad3 to transmit TGF-β1 signaling, and thereby inhibit filaggrin expression, and *FLG* rs75235053 C>G may add new splicing sites to reduce filaggrin monomers. It has been known that the level of Th2 cytokine IL-4 is increased in EA patients, and IL-4 increases airway epithelial permeability and enhances inflammatory response through some unclear mechanisms. To figure out whether filaggrin is involved in immune responses in asthma, we have treated human respiratory epithelial cell line BEAS-2B cells with IL-4 and found that the expression levels of filaggrin and E-cadherin decreased significantly in a time and dose-dependent manner, suggesting that IL-4 increased airway epithelial permeability by reducing filaggrin and adhesion molecule. In addition, in our study, IL-4 increased the expression of epithel-derived inflammatory cytokines IL-33 and TSLP which further enhanced the Th2 inflammatory response. To investigate the role of filaggrin in development of EA, knockdown filaggrin with siRNA revealed a decrease in E-cadherin levels, which were further down-regulated by IL-4 stimulation. Knockdown of filaggrin alone did not affect the levels of IL-33 and TSLP, but further exacerbated the decrease of IL-33/TSLP caused by IL-4, suggesting that filaggrin may involve in IL-4R signaling pathway to regulate the level of IL-33/TSLP. In conclusion, in the Th2 cytokine milieu of asthma, *FLG* deficient mutation in airway epithelial cells may increase the epithelial permeability and the expression of IL-33/TSLP which positively feedback the Th2 inflammation response.

## Introduction

Asthma is a heterogeneous disease in which genetics and the environment work together. In previous reports, it has been proved that epigenetics, respiratory infections, diet and other factors affect the onset of asthma. Genetic component is substantial, with estimates of heritability ranging from 35% to 95% in asthma (1). As of 2006, prior to publication of the first asthma genome-wide association study (GWAS), candidate gene studies had implicated more than 100 genes in the etiology of asthma (2). There are 38 loci or genomic regions available in GWAS Catalog associated with asthma nowadays (3). In the post-GWAS era, more studies focused on genomics related to asthma sub-phenotypes (4–6) or a certain clinical feature or asthma pharmacogenetics (7, 8). These studies can reduce heterogeneity, identify new asthma risk loci, even in smaller samples (9), and be more useful for elucidating the genetics and etiology of asthma.

Eosinophilic asthma (EA) refers to a clinical inflammatory phenotype of asthma with a significant number of sputum, airway, and/or blood eosinophils, and approximately half of asthmatics in this category. Recognizing EA is not only helpful for identifying patients who will benefit from inhaled corticosteroids (ICS), but also for identifying patients who will be suitable for the biologic therapies targeted eosinophilia and/or related inflammatory pathways in severe asthma patients (10). In our previous report, we described the clinical characteristics and biomarkers in different asthma subtypes. However, genomic analysis on EA has rarely been reported until now which need further exploration. Targeted next-generation sequencing (targeted region sequencing, TRS) technology combined microarray technology with next-generation sequencing has advantages of efficiency, accuracy, low-cost, high-coverage, and capability to discover new mutation sites. This technology has high clinical application values and is suitable for the detection of large number of clinical samples, and has been extensively used in tumor researches and clinical trials (11). Thus, we performed TRS on the peripheral blood of EA patients to determine the genomic variations [mainly including SNPs and Insertion-Deletions (InDels)] by comparing with healthy controls, analyzed their association with clinical characteristics and regulatory mechanisms to explore the molecular basis of the pathogenesis of EA. Among these screened SNPs, *FLG* rs192116923 T>G allele was found for the first time to be associated with EA patients with atopic dermatitis (AD). In addition, *FLG* rs75235053 C>G allele and *GSTM1* rs412302 G>A allele were associated with severe EA.

Filaggrin (FLG) mainly expresses in epidermal tissues and plays an important role in the formation of the stratum corneum and the maintenance of epithelial barrier. *FLG* loss-of-function mutations firstly found in ichthyosis vulgaris patients were related to AD or eczema, food allergy, asthma and hay fever (12). Recent GWAS reports found *FLG* loss-of-function mutations were closely related to childhood-onset and severity of asthma (13). It has been reported that filaggrin can be depressed by Th2 cytokines such as IL-4 which play an important role in the pathogenesis of asthma and AD (14). Besides, filaggrin deficiency has been proved being correlated with the increasing of the epithelial cell–derived cytokines, thymic stromal lymphopoietin (TSLP) and IL-33, which are critical mediators of T helper type 2 inflammation (15–17). In this report we demonstrated that filaggrin constitutively expressed in the human bronchial epithelial cell line BEAS-2B cells. To evaluate the function of filaggrin in bronchial cells upon the on-set of asthma, we simulated the cytokine environment in asthma with Th2 cytokine IL-4. Then we found that the expressions of filaggrin and E-cadherin were reduced while IL-33 and TSLP were increased by IL-4 inducing. Furthermore, *filaggrin* deficiency probably enhanced Th2 inflammatory response by reducing the expressing of E-cadherin and inducing the expression of IL-33 and TSLP with IL-4 inducing. Corresponding to the genetics analysis of EA patient, we conjectured that the loss-of-function mutation of *FLG* probably results in the reduction of the adhesion molecule E-cadherin and the secretion of type 2 cytokines (TSLP and IL-33) in bronchial epithelial cells which may result in the on-set or exacerbation of asthma.

## Methods and Materials

### Patient Selection

From November 2016 to May 2019, 77 EA patients were recruited from the Department of Respiratory, Chinese PLA general hospital, Beijing. In addition to meeting the asthma criteria in Global Initiative for Asthma (GINA) 2020 (18), patients should also meet the following inclusion criteria: The percentage of eosinophils in induced sputum is ≥ 3%, and the percentage of neutrophils in induced sputum is ≤61% (19); and/or the blood eosinophils count is ≥0.3×10^9^/L; and/or fractional exhaled nitric oxide (FeNO) is ≥35ppb (20). Patients with acute infections, other respiratory diseases, tumors, liver and kidney dysfunction, etc. should be excluded. The present study was approved by the Human Research Ethics Committee of the Chinese PLA general hospital, and all participants provided written informed consent.

### Observable Clinical Characteristics and Definitions

(i) Early-onset (<12 years) and late-onset (≥12 years) EA (21). (ii) Allergic [allergic sensitization and symptoms in response to allergen exposure (allergic sensitization: specific IgE demonstrated by skin prick-puncture tests or specific IgE *in vitro* tests to at least one of an appropriate panel of allergens)] (22) and non-allergic EA. (iii) Frequent exacerbations (with 2 or more asthma exacerbations leading to emergency room visit or hospitalization within the last 12 months) (23) and infrequent exacerbations of EA. (iv) Combined AD [also known as atopic eczema, eczema, which often occur in families with atopic diseases (atopic dermatitis, asthma, and/or allergic rhino-conjunctivitis). The clinical dermatologists confirmed the diagnosis of AD] (24). (v) Severe [high dose of ICS plus a second controller (and/or systemic corticosteroids) required to prevent asthma from becoming uncontrolled or still uncontrolled] (25) and mild-moderate eosinophilic asthma [well controlled with GINA (18) Step 1-3 treatment with as-needed reliever medication monotherapy or low dose of controller treatment, such as ICS, leuko-triene receptor antagonists (LTRA)].

### Target Region Sequencing

Target Region Sequencing (TRS) is a technique of next generation sequencing (NGS) which captures targeted genes or specific regions of the genome. In this study, genomic DNA samples were extracted from peripheral blood of 77 EA patients. An asthma disease probe (Agilent, USA) is 491.178 kb, designed to capture the exon regions (including the 10 bp flanking on either side) and upstream regions (1 kb) of 109 asthma candidate genes (**Table S1**) frequently involved in literatures including several large-scale GWAS studies. Samples with index tag were pair-end sequence simultaneously on Illumina HiSeq 2500 Sequencer (Illumina, San Diego, CA) for 150 cycles. FASTQ sequencing files were aligned with human genome assembly GRCh37 using Burrows-Wheeler Aligner (BWA,26), processed using Picard to mark duplicates package and utilizing GATK 3.0 Haplotype Caller to call the raw variants. The data received had an average read depth of more than 300-fold coverage in more than 98% of the targeted regions.

### Variation Filtering and Annotation

Based on the alignment results, SAM tools were applied to identify SNP sites. SNPs were annotated using ANNOVAR software. The public databases, including the dbSNP147 database, 1000 Genome Project, NHLBI-ESP Project (esp6500si), ClinVar and GnomAD (The Genome Aggregation Database), were used to screen the variants. The functional effect prediction was evaluated using SIFT, Polyphen 2, Mutation Taster, CADD score. InDel mutations in gene coding or splicing regions were likely to alter protein translation. Non-integer three-fold frameshift InDel mutations may cause changes in the entire reading frame and have an impact on gene function. Filtering and annotation methods are roughly the same as SNP. All variants were assigned in American College of Medical Genetics and Genomics standards and guidelines (26).

### Bioinformatics Analysis

Polyphen 2, SIFT score, Mutation Taster, and CADD were used to predict whether the SNP was harmful mutation. HaploReg v4.1 (https://pubs.broadinstitute.org/mammals/haploreg/haploreg.php), Regulome RB online analysis (http://www.regulomedb.org/), SNPinfo (https://snpinfo.niehs.nih.gov/) and SNP2TFBS (https://ccg.epfl.ch/snp2tfbs/snpviewer.php) were used to predict the gene regulation or biological function of SNPs. Alamut Visual 2.12 software was used to predict the effect of SNPs on splicing of precursor mRNA.

### Cell Culture and Cell Induction Conditions

Immortalized human keratinocyte HaCaT cells and BEAS-2B (human bronchial epithelium cells transformed with Ad12-SV40 2B) cells (Fenghui Bio Inc., Hunan, China) were cultured in DMEM medium (HyClone, China) containing 10% fetal bovine serum (Gibco, USA), 100 U/ml penicillin, and 100 μg/ml streptomycin (Solarbio, China), at 37°C with 5% CO2 and 95% humidity. The experimental cells were divided into the control group (phosphate buffer saline (PBS), Solarbio), IL-4 (R&D Systems China) 25 ng/ml, 50 ng/ml, 100 ng/ml induction group and the induction duration was 12h, 24 h and 48h, respectively (27). In the stimulation experiments, cells were cultured in serum-free medium.

### Knockdown of Filaggrin Using siRNA Transfection

Three sequences of filaggrin siRNA and a siRNA negative control were designed and synthetized, as follows: siRNA-307 (sense GCACAGUCAUCAUGAUAAACA, antisense UUUAUCAUGAUGACUGUG CUU), siRNA-485 (sense GGAUAUUCACCUACUCAUAGA, antisense UAUGAGUAGGUGAAUAUCCU U), siRNA-5581 (sense CAGAAGUGUCAGCAGACAAAC, antisense UUGUCUGCUGACACUUCUGGA), and siRNA-NC (sense UUCUCCGAACGUGUCACGUTT, antisense ACGUGACACGUUCGGAGAATT). BEAS-2B cells were selected with good growth status, and were inoculated into 6-well plate, adjusting the cells to 2×10^5^ per well. A mixture of 5 μL lipofectamine 6000TM (Beyotime, China), 100 pmol siRNA and 125 μl DMEM serum-free medium (HyClone) were added and incubated for 20 min at room temperature. Then each well was added with 250 μl siRNA-Lipo6000TM mixed culture medium. Six hours after transfection, the medium in the wells was replaced with DMEM complete medium, and the culture was continued for 48 h. Then cells were incubated with IL-4 or control for another 24 hours and then collected for analysis.

### Real-Time Quantitative RT-PCR Analysis

Real-time quantitative RT-PCR was performed to quantitatively estimate the mRNA expression of filaggrin and measure the filaggrin knock-down efficiency, and estimate the mRNA expression of TSLP, IL33, filaggrin, and E-cadherin. Total RNA from cultured HaCaT and BEAS-2B cells was isolated using Trizol reagent (Invitrogen) according to the published method. The purified RNA was reverse transcribed to cDNA by using the PrimeScript RT reagent Kit (Takara) according to the manufacturer’s instruction. MA-686 Real-time PCR Instrument (Molarray, Suzhou, China) was used to analyze quantitative RT-PCR according to the manufacturer’s instruction. Primer sequences were as follows: Filaggrin: forward: 5’-CTCATCACAGCCACACCACATCC-3’, reverse: 5’-GCCGTCTCCTGATTGTTCATCGT TA-3’; TSLP: forward: 5’-GCTACTCAGGCAATGAAGAAGAGGAG-3’, reverse: 5’-CCTTGTAATTGTGACAC TTGTTCCAGAC-3’; IL33: forward: 5’-GGCATTCTTACACGAGGAAGTGAAGT-3’, reverse: 5’-TGTCTCATC TAGGCTCTGGTAGGTT-3’; E-cadherin: forward: 5’-TCCAGCTTCACCATCAAAGTT-3’, reverse: 5’-TTCCACCA CGATCTCATACCT-3’; GAPDH: forward: 5’-TCAAGAAGGTGGTGAAGCAGG-3’, reverse: 5’-GCGTCAAA GGTGGAGGAGTG-3’. The reaction system was: 2× Real-time PCR Master Mix 10 μL, 20 μM primers 0.8 μL, cDNA template 2 μL, 5 U/μl rTaq DNA polymerase 0.4 μL and ddH2O 6.8 μL. The following PCR condition were used: 95°C for 3 min, 95°C for 30 s, 55°C for 20 s, 40 cycles, and 72°C for 20 s. This experiment was carried out in triplicate and independently repeated at least three times. The expression levels were normalized to an endogenous control, GAPDH. The formula RQ = 2-ΔΔCt was used to calculate, and 2-ΔΔCt was used to express the difference in gene transcript expression.

### Western Blotting Analysis

Whole BEAS-2B cell extracts were prepared in RIPA lysis buffer (Beyotime) for Western blotting and centrifuged at 14,000 rpm for 15 min at 4°C. The supernatants were then obtained and protein concentrations were detected using the BCA protein quantification kit (Beyotime). The protein extracts were loaded and separated by SDS-PAGE and then transferred to PVDF membranes (Millipore). The membranes were incubated overnight at 4°C with specific primary antibodies. Then, membranes were incubated with secondary antibodies and visualized using the Tanon 5200 automatic chemiluminescence image analysis system (Science Technology Co., LTD). The primary antibodies used for immunoblotting were as follows: E-cadherin (1:500, Cell Signaling Technology), filaggrin (1:500, Affinity), IL-4R (1:500, Affinity)GAPDH (1:5000, Cell Signaling Technology).

### Immunofluorescence Staining

Cells cultured were fixed with 4% paraformaldehyde and kept overnight at 4°C, permeabilized with PBS containing 10% goat serum for 1 h at room temperature to block non-specific protein interactions. Rabbit anti-filaggrin primary polyclonal antibody (1: 500, Abcam) was diluted with PBS at 4°C overnight. The cells were washed in PBS and then incubated with 594-conjugated AffiniPure donkey anti-rabbit secondary antibody IgG (H+L) (Jackson, 1:300) at 4°C overnight. After washing the cells with PBS, DAPI nuclear staining was performed for 3 min. After PBS was washed again, the cells were imaged using confocal microscopy (Leica Microsystems, Wetzlar, Germany).

### Immunohistochemistry

The paraffin sections of human normal bronchia, lung and skin were provided by the department of pathology, 8th Medical Center of Chinese PLA General Hospital with the approval of the patients. The slices were processed as the protocol previously described (28) and then dipped into rabbit anti human filaggrin antibody (Affbiotech, Jiangsu, China) for over 2 hours at room temperature. Rabbit IgG before immunization and antigen absorbent immune serum were applied as negative controls. After three washes in PBS, the slices were incubated with HRP-conjugated goat anti-rabbit IgG (ZSGB-BIO, Beijing, China) for 30 min at room temperature. Then antibody complexes were visualized by incubation with DAB chromogen. As the protocol, sections were counterstained with Mayer’s hematoxylin for 10 sec, dehydrated through gradient ethanol, cleared in dimethyl benzene, mounted, and examined using light microscopy (Olympus,Tokyo,Japan).

### ELISA

Concentrations of released IL-33 and TSLP in the cell supernatants were assessed. We used the human IL-33 ELISA Kit (Meimian Biology, China) and the human TSLP ELISA kit (Meimian Biology, China). Standard reagents were prepared according to the instructions, and the standard dilutions and cell culture supernatant were added to the detection wells. The diluted antibody was added and incubated at room temperature for 2 h. Labeled streptavidin (1:100 dilution) 100 μl and chromogenic substrate 100 μl were added to each well, and incubated in the dark for 30 min. Within 30 minutes after the color change, a microplate reader (BioTek Epoch, USA) was used to detect the dual wavelength and the optical density (OD) value at the reference wavelength was determined. Regression fitting was used to generate a standard curve, and the OD value was linearly analyzed.

### Statistical Analysis

Statistical analysis was performed using SPSS 20.0(SPSS Inc., Chicago, IL, USA) and GraphPad Prism 8.0 software (GraphPad Software Inc., San Diego, CA, USA). On the SHEsis platform (http://analysis.bio-x.cn/myAnalysis.php), Hardy-Weinberg equilibrium test was performed on the gene distribution of each SNP locus in 77 patients and 431 healthy controls in Chinese internal database, and linkage disequilibrium between SNPs was also detected. Data were expressed as the mean ± standard deviation (SD) with normal distribution or median and interquartile range (IQR) without normal distribution. Significance of differences between the groups was evaluated with independent samples Student’t t -testand LSD analysis was used for multiple comparisons between the two groups. The Pearson chi-square test or Fisher’s exact test was used for the comparison between two categorical variables. All statistical tests were set to two-way test with a test level of α=0.05.

## Results

### Differential SNPs Detected by Case-Control Association Analysis of EA Patients

Clinical baseline data and classified clinical characteristics distribution of EA patients were shown in **Table 1**. The median age of EA patients was 48 (from 36 to 54) years-old and there were 38 (49.4%) female patients. There was no significant difference in the median age and sex ratio of EA group and healthy control group. The proportion of patients with a history of smoking was 42.9%. The percentage of patients with allergic rhinitis (AR) and AD was 68.8% and 37.7%, respectively. The median duration of asthma or wheezing at baseline was 9 (from 3 to 19) years. The number of early- and late-onset EA patients was 16 and 61, respectively. All of the early-onset EA patients were also allergic. The number of allergic and non-allergic EA patients was 70 and 7, respectively. And the number of mild-moderate and severe EA patients was 20 and 57, respectively. Moreover, the median frequency of asthma exacerbations in the past 1 year was 2, while the median asthma control test (ACT) score was 17. There were 32 and 45 EA patients with frequent and infrequent exacerbations, respectively. The number of EA patients with and without AD was 29 and 48, respectively. According to the laboratory data, the median FeNO and total serum IgE was 44 (25-75) ppb and 229 (88.7-590) IU/ml, respectively. The median percent of peripheral blood eosinophils was 4.8% (3.5%-8.9%). The median percent of induced sputum eosinophils was 3% (0.5%-8%).

**Table 1:** The clinical baseline data of EA patients.

Clinical data	n = 77	n = 431
Female, n(%)	38 (49.4)	220 (51.0)
Age (years), median (IQR)	48 (36–54)	45 (31–55)
BMI (kg/m^2^), median (IQR)	23.80 (21.09-26.90)	22.5(20.11-25.20)
Duration of asthma (years), median (IQR)	9 (3-19)	
Smoking status, n (%)		
Never	44 (57.1)	
Former or current	33 (42.9)	
Family history of asthma, n (%)	48 (62.3)	
Comorbidities, n (%)		
Allergic rhinitis	53 (68.8)	
Atopic dermatitis	29 (37.7)	
Chronic sinusitis	29 (37.7)	
Sinus polyposis	12 (15.6)	
ACT score, median (IQR)	17 (15-20)	
Asthma exacerbations in past one year, median (IQR)	2 (2-3)	
FEV1% (FEV1% predicted), mean ± SD	72.82 ± 23.06	
Postbronchodilator FEV1/FVC % pred, mean ± SD	68.45 ± 14.57	
Induced sputum eosinophils* (%), median (IQR)	3 (0.5-8)	
Blood eosinophils number (*10^6), median (IQR)	399.04 (226.35-587.05)	
Blood eosinophils (%), median (IQR)	4.8 (3.5-8.9)	
FeNO (ppb), median (IQR)	44 (25-75)	
Total IgE (IU/mL), median (IQR)	229 (88.7-590)	

*Induced sputum eosinophils % value was available in 61 (79.2%) patients.

BMI, body mass index; ACT, asthma control test; FEV1, forced expiratory volume in the first second; FVC, forced vital capacity.

In association analysis of allele frequencies of 77 EA patients and 431 healthy individuals in Chinese internal database excluding sites that did not meet the H-W equilibrium, we detected 63 single nucleotide variants (SNVs). Of these variants, we detected 28 promoter variants, 34 exonic variants, 1 splicing variant. Four of them were newly discovered SNPs. Meanwhile, seven missense SNPs (*ADAM8* rs553707790, *TGFB1* rs943475580, *NLRP3* rs188623199, *IL25* rs770223596, *ATG7* rs555273544, *IRF* c.G628A, *IL13* rs770488630) were most likely pathogenic variants. A small number of patients (less than 4) were selected out for mutation sites, and 41 SNPs were obtained (**Table 2**). These SNPs had linkage disequilibrium: *HLA-G* rs3823321 and rs14722477, *HLA-G* rs1130356 and rs1049033, *CCL11* rs17735961 and rs1129844, *SPINK5* rs2303063 and rs2303062, *MS4A2* rs1441585, rs574700 and rs569108, *CCL2* rs2857656 and rs4586 (r^2^≥0.8). After all, all the sequencing data of the 77 EA patients have been uploaded to GenBank with the Bioproject ac number PRJNA718105 (https://dataview.ncbi.nlm.nih.gov/object/PRJNA718105?reviewer=djfq3k2vpjeb5uvi8loaag0d3f).

**Table 2:** Screened SNP list of EA patients.

Gene	Av SNP	Function	REF	ALT	Alt(shared_het)N.	P_value	OR	Ref rate
*CCL11*	rs4795896	promoter	T	C	68(33)	0.003	1.739	0.572
*CCL11*	rs17735961	promoter	C	A	10(9)	0.008	0.432	0.136
*CCL11*	rs1129844	exonic missense	G	A	10(9)	0.008	0.428	0.136
*CCL2*	rs2857656	promoter	G	C	67(32)	0.008	1.625	0.570
*CCL2*	rs4586	exonic synonymous	T	C	67(32)	0.010	1.597	0.570
*IL-2*	rs2069763	exonic synonymous	C	A	64(38)	0.036	1.452	0.515
*CHI3L1*	rs10399805	promoter	G	A	48(33)	0.004	1.684	0.314
*HLA-G*	rs1736937	promoter	C	A	44(28)	0.050	1.430	0.301
*HLA-G*	rs3823321	promoter	G	A	23(22)	0.028	0.600	0.233
*HLA-G*	rs1130356	exonic synonymous	C	T	34(26)	0.007	1.765	0.167
*HLA-G*	rs1049033	exonic synonymous	C	T	31(25)	0.028	1.616	0.156
*HLA-G*	rs12722477	exonic missense	C	A	23(22)	0.035	0.603	0.229
*CXCL1*	rs2071425	exonic synonymous	A	G	41(32)	0.040	0.681	0.391
*CXCL1*	rs74544699	promoter	A	G	29(26)	0.007	1.850	0.137
SPINK5	rs2303063	exonic missense	G	A	46(45)	0.006	0.590	0.451
SPINK5	rs2303062	splicing	A	G	45(45)	0.003	0.567	0.451
*FLG*	rs192116923	exonic missense	T	G	33(27)	0.025	1.601	0.213
*FLG*	rs75448155	exonic missense	C	T	38(38)	0.000	3.138	0.131
*FLG*	rs12742178	exonic synonymous	G	A	32(32)	0.000	2.814	0.142
*FLG*	rs75235053	exonic missense	C	G	27(27)	0.003	2.169	0.101
*FLG*	rs147613842	exonic missense	T	C	6(5)	0.029	2.880	0.024
*FLG*	rs200249011	exonic missense	C	T	4(4)	0.007	9.026	0.005
TRPV2	rs1441585	promoter	T	C	32(26)	0.010	1.748	0.165
MS4A2	rs574700	promoter	C	T	28(22)	0.045	1.552	0.163
MS4A2	rs569108	exonic missense	A	G	32(26)	0.007	1.790	0.163
ADAM33	rs2271511	exonic synonymous	C	T	32(31)	0.028	1.647	0.150
CTLA4	rs16840252	promoter	C	T	26(23)	0.041	1.615	0.133
CTLA4	rs5742909	promoter	C	T	24(21)	0.462	1.566	0.128
GSTM1	rs36209093	promoter	C	T	22(18)	0.007	2.088	0.187
GSTM1	rs412302	promoter	G	A	20(19)	0.017	0.548	0.213
TNFSF10	rs9848839	promoter	T	C	21(21)	0.016	2.011	0.087
TNFSF10	rs766827283	promoter	T	C	16(16)	<0.001	19.908	0.008
TNFSF10	rs751868102	promoter	T	C	5(5)	<0.001	32.659	0.002
PLA2G7	rs1805017	exonic missense	C	T	17(16)	0.014	0.527	0.184
*LTC4S*	rs3776944	promoter	G	A	13(11)	0.030	0.543	0.157
TRPV1	rs375458057	exonic synonymous	A	C	10(9)	0.034	2.371	0.032
TBX21	rs2074190	exonic synonymous	A	G	10(10)	0.029	0.474	0.109
*MTOR*	rs17848553	exonic synonymous	G	A	10(10)	0.032	2.323	0.039
MTOR	rs17036536	exonic synonymous	C	G	10(10)	0.032	2.323	0.038
DPP4	rs115498481	promoter	G	T	9(9)	0.039	2.367	0.024
TLR4	rs75048248	promoter	C	T	7(7)	0.006	4.503	0.017

Alt N., number of mutation patients; shared_het, number of patients with heterozygous mutations at the current site. OR, odds ratio.

### Correlation Between Genomic Variations and Clinical Characteristics of EA Patients

Correlation analysis was performed between genomic variations and clinical characteristics of EA patients. There were several SNPs associated with clinical characteristics of categorical and continuous variables of EA patients, shown in **Table 3**. *FLG* rs75235053 C>G allele (P=0.032, OR 4.121 comparing CG and CC genotype) and *GSTM1* rs412302 G>A allele (P=0.016, OR 9.500 comparing AA+AG and GG genotype) were associated with severe EA. Besides, another *FLG* mutation *FLG* rs192116923 T>G allele was associated with EA patients with AD (P <0.001, OR 5.983 comparing GT + GG and TT genotype). Besides, *SPINK5* rs2303063 G>A allele was linked to early-onset allergic EA (P=0.011, OR 6.344 comparing GA and GG genotype), and *TLR4* rs75048248 C>T allele was associated with frequent-exacerbation EA (P = 0.018, OR 10.154 comparing CT and CC genotype).

**Table 3:** SNPs associated with clinical characteristics of EA patients.

SNP	Clinical characteristics	Alt N./value(N)	Wild N./value(N)	P_value	OR	OR 95%CI
*SPINK5* rs2303063 G >A	Early-onset; Late-onset	14; 32	2; 29	0.011	6.344	1.327-30.325
*TLR4* rs75048248 C >T	Frequent; infrequent	6; 1	26; 44	0.018	10.154	1.157-89.091
*FLG* rs75235053 C >G	Mild -moderate; severe	3; 24	17; 33	0.032	4.121	1.084-15.665
*GSTM1* rs412302 G >A	Mild -moderate; severe	1; 19	19; 38	0.016	9.500	1.181-76.418
*FLG* rs192116923 T >G	With AD; No AD	20; 13	9; 35	<0.001	5.983	2.175-16.460
*CTLA4* rs5742909 C>T	Total IgE	835.78 ± 1061.71(24)	423.38 ± 624.85(53)	0.036	–	–
*GSTM1* rs412302 G>A	Blood eosinophil count	697.90 ± 647.63(20)	415.15 ± 34.10(57)	0.047	–	–
*LTC4S* rs3776944 G>A	Blood eosinophil %	4.21 ± 2.28(16)	6.99 ± 4.66(61)	0.001	–	–

We also indentified statistically significant associations of some SNPs and blood eosiniphil count and IgE level. Among them, *CTLA4* rs5742909 C>T allele was positively correlated with serum total IgE of EA patients (P =0.036), *GSTM1* rs412302 G>A allele was positively correlated with blood eosinophil count of EA patients (P =0.047), and *LTC4S* rs3776944 G>A allele was negatively correlated with blood eosinophil percentage of EA patients (P =0.001).

### Bioinformatics Analysis of the Biological Function of *FLG* exon SNPs

Since *FLG* rs75235053 C>G and rs192116923 T>G were associated with severe EA and EA patients with AD, respectively, their biological functions were tested by bioinformatics. *FLG* encodes the filaggrin precursor protein profilaggrin, which contains 4061 amino acids. Its protein code is mainly in the third exon region, and mutations are also mainly distributed in this region. Rs192116923 T >G is a missense mutation p. Glu 2652 Asp, while rs75235053 C >G is a missense mutation p. Ser 3662 Thr, and both of them are anticipated by Polyphen 2, SIFT score, Mutation Taster, and CADD as non-harmful mutations. HaploReg v4.1 (https://pubs.broadinstitute.org/mammals/haploreg/haploreg.php) predicts that rs192116923-T mutation to -G can modify its binding motif to Smad3, and SNP2TFBS (https://ccg.epfl.ch/snp2tfbs/snpviewer.php) predicts that it can bind to Smad2_Smad3_Smad4, whereas PROMO suggests that rs75235053-C mutation to -G may reduce the binding to c-Jun.

### IL-4 Decreased the Expression of Filaggrin and E-Cadherin, but Increased the Expression of IL-33 and TSLP in Time- and Dose-Dependent Manner

**Figure 1:**
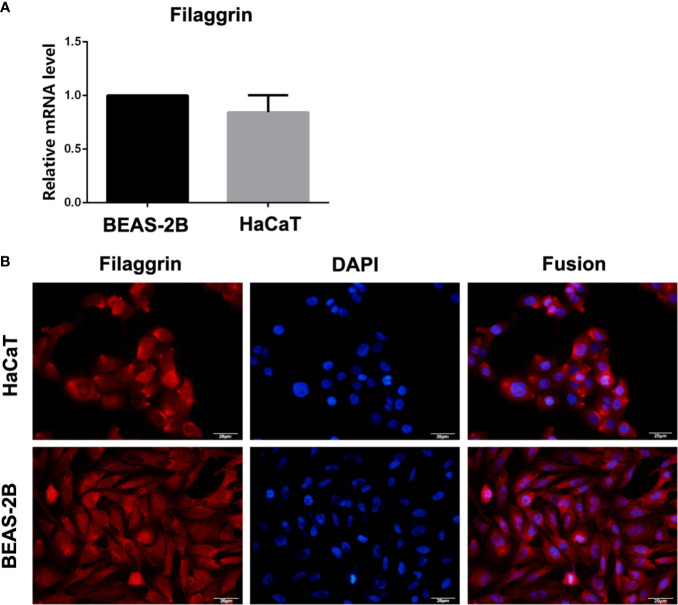
The mRNA and protein expression of filaggrin in BEAS-2B and HaCaT cells. **(A)** Filaggrin mRNA expression in HaCaT cells and BEAS-2B cells. **(B)** Immunofluorescence (IF) results of filaggrin in BEAS-2B and HaCaT cells. The photographs were magnified of 400 times and the bars represented 20μm.

**Figure 2:**
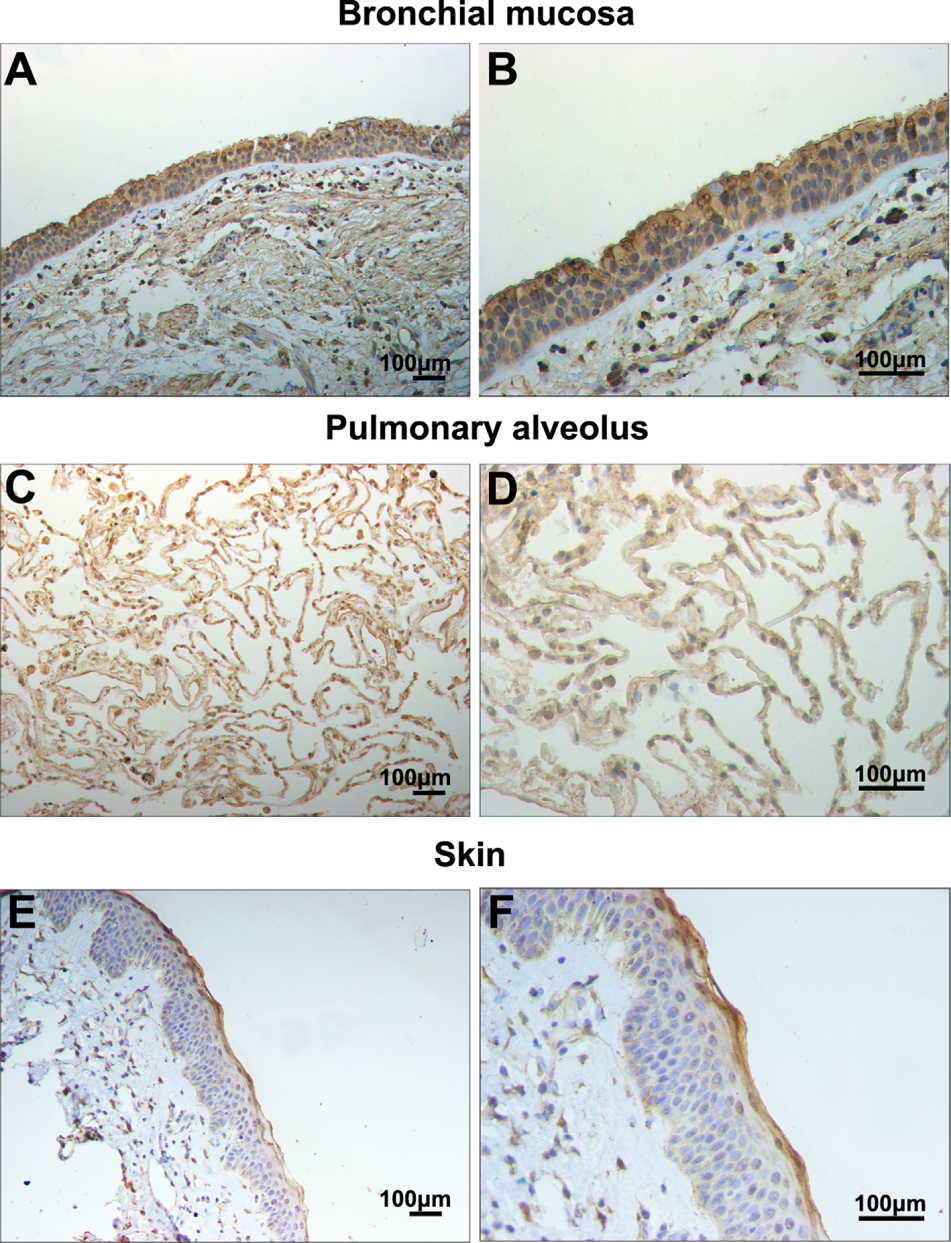
Filaggrin was expressed in normal human bronchial epithelial cells and alveolar epithelial cells. Identification of filaggrin expression in normal human bronchial mucosa (representative example of n=6), pulmonary alveolus (representative example of n=5) and skin tissues (representative example of n=3) by immunohistochemistry (IHC). Magnification of **(A, C, E)**:100×; **(B**, **D**, **F)**:200×. Bar=100μm.

In this study, filaggrin has been proved constitutively expressed in human bronchial epithelial cell line BEAS-2B cells *via* RT-PCR and IF detection, and mainly distributed in the cytoplasm (**Figure 1**). Most importantly, in this study, it has been confirmed that filaggrin highly expressed in human normal bronchial epithelial cells and relatively lowerly expressed in alveolar epithelial cells *in vivo* (**Figure 2**).

**Figure 3:**
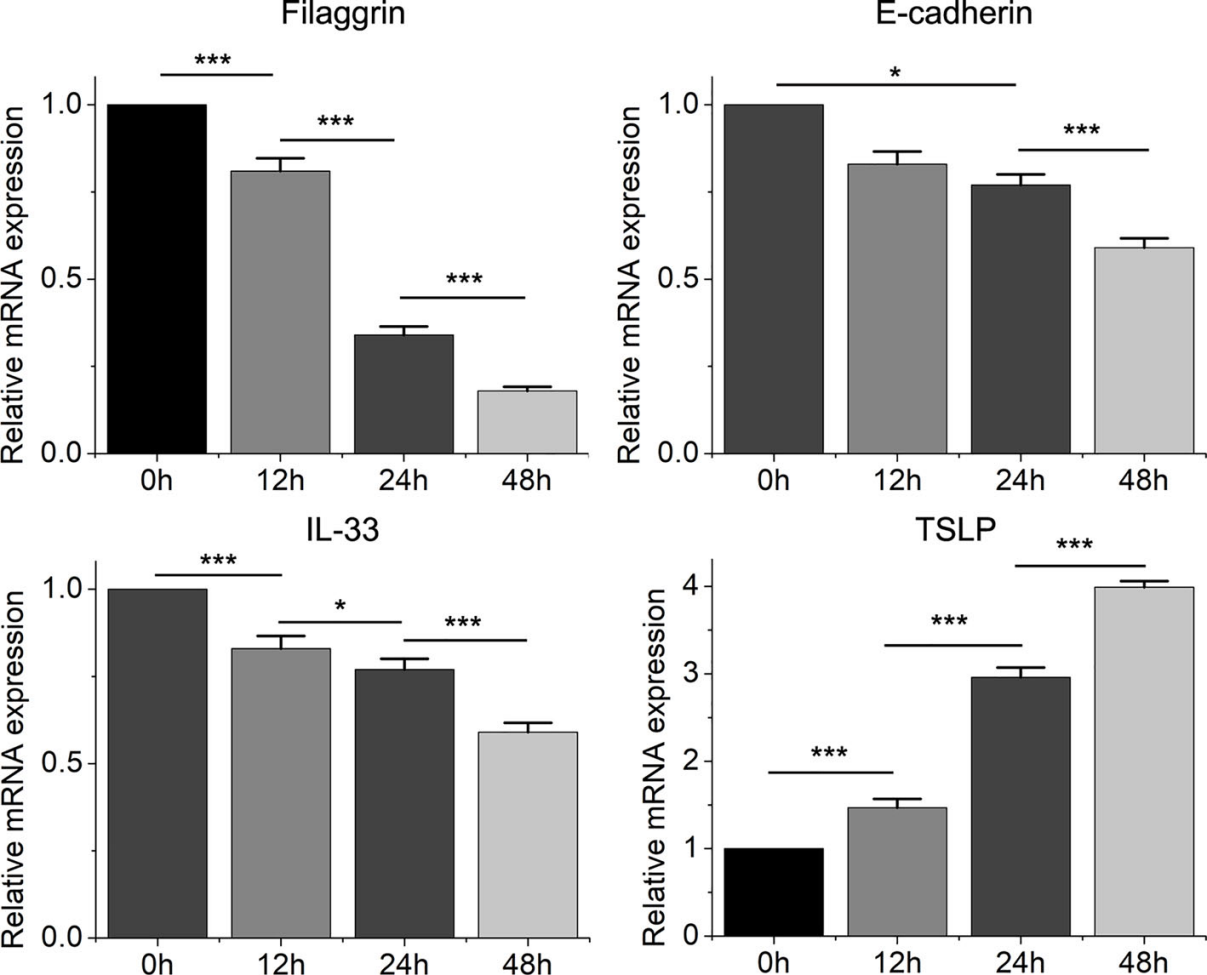
The mRNA expression of filaggrin, E-cadherin, IL-33 and TSLP in BEAS-2B cells with IL-4 treatment for different periods. Quantitative data are presented as the mean ± standard deviation (n=3). The Student’s t-test was used to analyze the difference between groups. *P < 0.05 was considered as statistically significant. ***P < 0.001.

**Figure 4:**
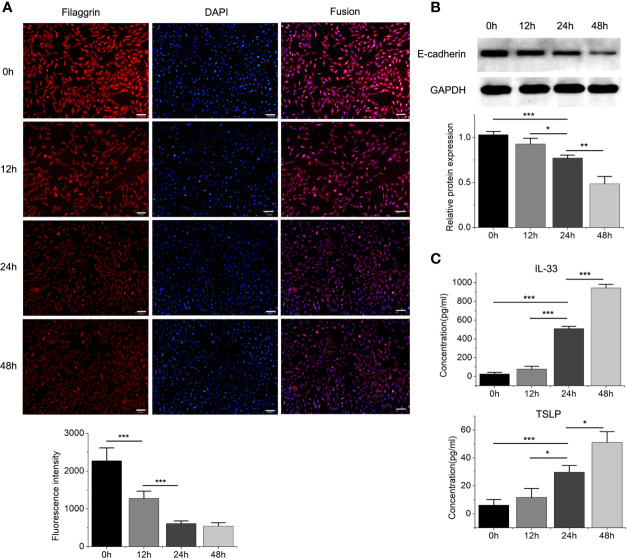
The protein expressions of filaggrin, E-cadherin, TSLP and IL-33 of BEAS-2B cells with IL-4 incubating for different periods. **(A)** The protein expression of filaggrin in BEAS-2B cells treated with IL-4 for different periods was detected by Immunofluorescence. The photographs were magnified of 200 times and the bars represented 50μm. **(B)** The protein expression of E-cadherin in BEAS-2B cells treated with IL-4 for different periods was detected by Western-blot. **(C)** IL-33 and TSLP in the cell culture supernatants with IL-4 treatment for different periods were detected by ELISA. Each experiment has been repeated for 3 times and presented as the mean ± SD (n=3). The Student’s t-test was used to analyze the difference between groups. *P < 0.05 was considered as statistically significant. **P < 0.01, ***P < 0.005.

**Figure 5:**
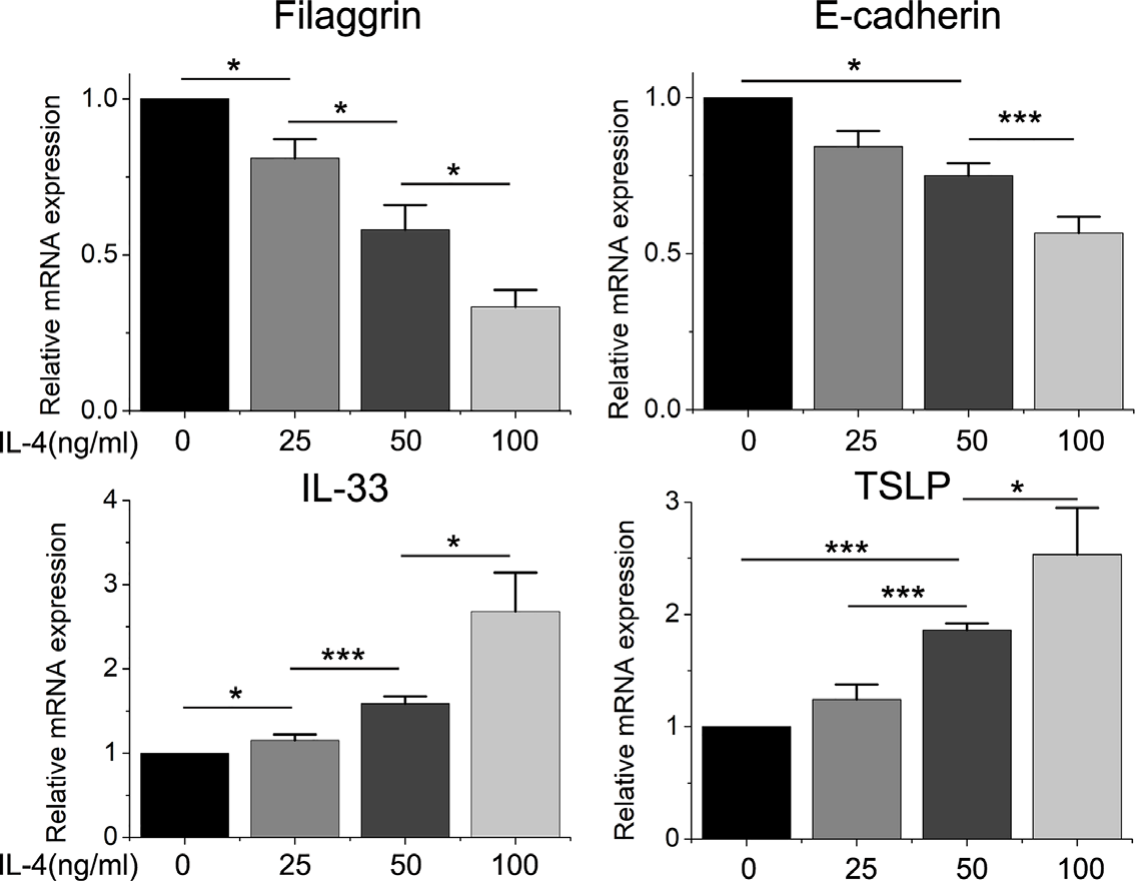
The mRNA expression of filaggrin, E-cadherin, IL-33 and TSLP in BEAS-2B cells treated with different concentration of IL-4. Quantitative data are presented as the mean ± standard deviation (n=3). The Student’s t-test was used to analyze the difference between groups. *P < 0.05 was considered as statistically significant. ***P < 0.005.

**Figure 6:**
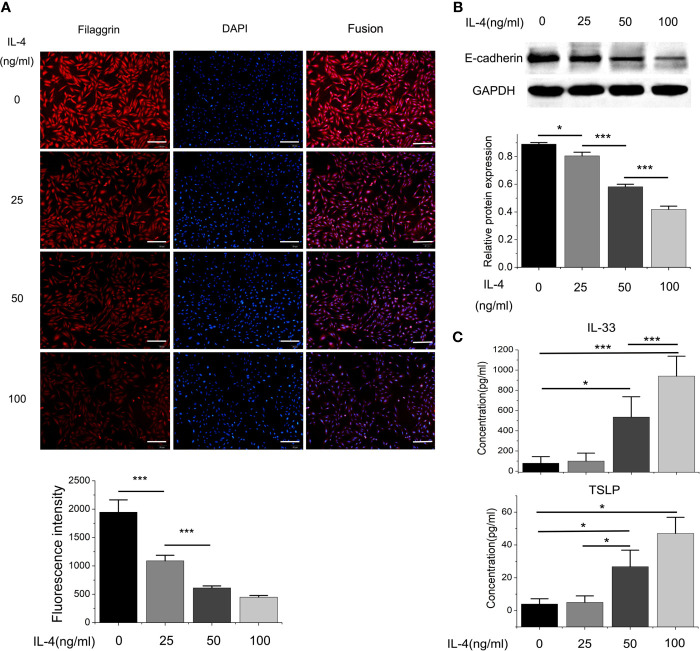
The protein expressions of filaggrin, E-cadherin, TSLP and IL-33 of BEAS-2B cells with different concentration of IL-4 incubation. **(A)** The protein expression of filaggrin in BEAS-2B cells treated with different concentration of IL-4 was detected by Immunofluorescence. The photographs were magnified of 200 times and the bars represented 50μm. **(B)** The protein expression of E-cadherin BEAS-2B cells treated with different concentration of IL-4 was detected by Western-blot. **(C)** IL-33 and TSLP in the cell culture supernatants with different concentration of IL-4 treatment were detected by ELISA. Each experiment has been repeated for 3 times and presented as the mean ± SD (n=3). The Student’s t-test was used to analyze the difference between groups. *P < 0.05 was considered as statistically significant. **P < 0.01, ***P < 0.005.

Then we found that the Th2 cytokine IL-4 reduced the mRNA and protein expressions of filaggrin and E-cadherin in BEAS-2B cells. E-cadherin is the main adhesion junction protein of airway epithelium. Moreover, filaggrin and E-cadherin were gradually down-regulated as the stimulation period prolonged (**Figures 3**, **4**) or the concentration of IL-4 increased (**Figures 5** and **6**). In **Figures 3** and **4**, it was shown that the expression of E-cadherin was not significantly increased until 24h incubation of IL-4 and the protein expression of filaggrin was not statistically change after 24h incubation of IL-4. Thus we chose 24h incubation period for the next step of experiments.

Different dose of IL-4 incubation also led to different results. In **Figures 5** and **6**, the protein expression of IL-33 and TSLP were statistically up-regulated until the IL-4 concentration rose to 50ng/ml, while there was no significant difference between the 50ng/ml IL-4 group and 100ng/ml IL-4 group, so that we chose the 50ng/ml of IL-4 in further researches.

On the other hand, the mRNA levels of epithelial cytokines IL-33 and TSLP, which are key regulators of Th2 inflammatory response, in the BEAS-2B cells with IL-4 inducing were obviously higher than those in the control group. Furthermore, IL-4 also increased the concentrations of soluble IL-33 and TSLP in BEAS-2B cells culture medium, and the regulation of IL-4 to IL-33 and TSLP was also dose- and time-dependent (**Figures 3**–**6**).

### Knockdown of Filaggrin Further Down-Regulated the Expression of E-Cadherin in BEAS-2B Cells, and Also Further Up-Regulated the Expression of IL-33 and TSLP After IL-4 Inducing

**Figure 7:**
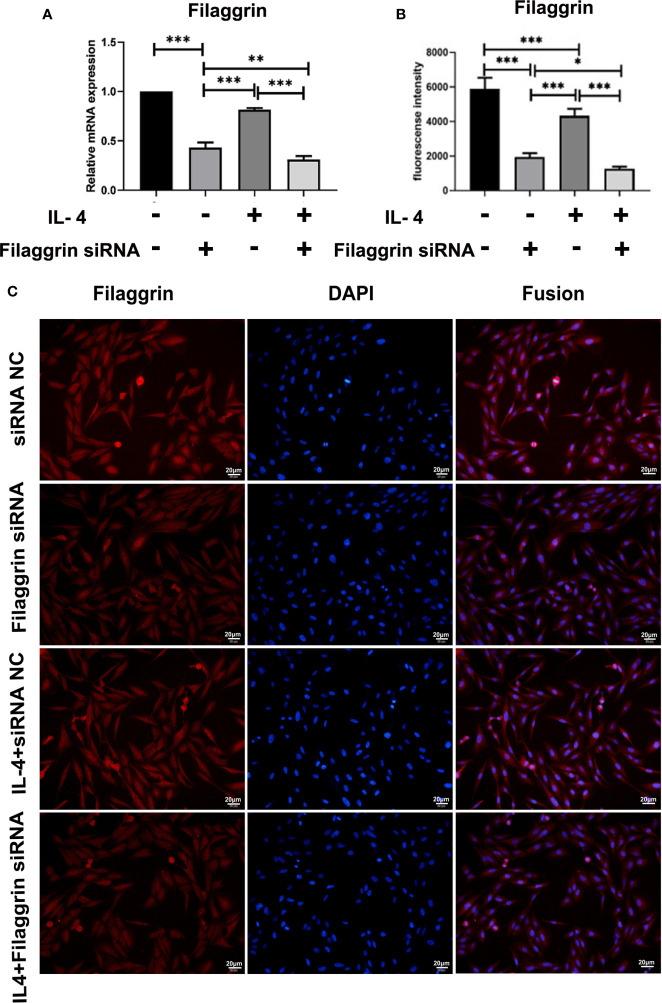
Filaggrin was more significantly decreased by IL-4 treatment after knock-down of filaggrin with specific siRNA. BEAS-2B cells were divided into four groups: siRNA NC group (control group), filaggrin siRNA group, IL-4 inducted group and filaggrin siRNA + IL-4 inducted group. **(A)** The mRNA expression of filaggrin among the four groups detected with RT-PCR. **(B, C)** The protein levels of filaggrin in the four groups were detected with IF. The photographs were magnified of 400 times and the bars represented 20μm. Each experiment has been repeated for 3 times and presented as the mean ± SD (n=3). The Student’s t-test was used to analyze the difference between groups. *P < 0.05 was considered as statistically significant. **P < 0.01, ***P < 0.005.

**Figure 8:**
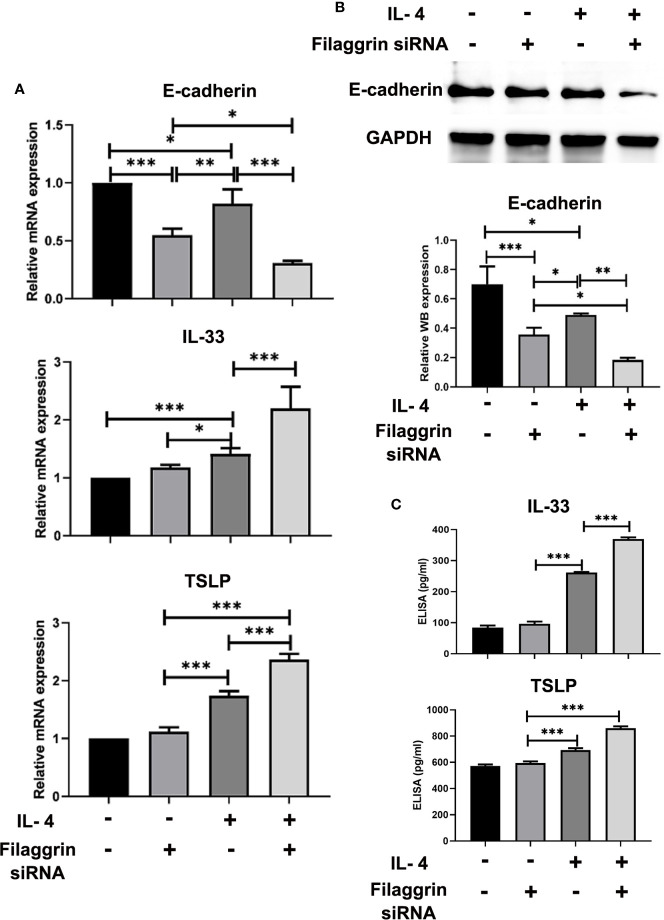
The expressions of E-cadherin, TSLP and IL-33 were more significantly modulated by IL-4 treatment after knock-down of filaggrin with specific siRNA. As same as the description in previous figure legend, BEAS-2B cells were divided into four groups: siRNA NC group (control group), filaggrin siRNA group, IL-4 inducted group and filaggrin siRNA + IL-4 inducted group. **(A)** The mRNA expression of E-cadherin, IL-33 and TSLP among the four groups detected with RT-PCR. **(B)** The protein levels of E-cadherin in the four groups were detected with Western-blot. **(C)** Levels of IL-33 and TSLP in the cell culture supernatant of the four groups were measured by ELISA. Each experiment has been repeated for 3 times and presented as the mean ± standard deviation (n=3). And the Student’s t-test was used to analyze the difference between groups. *P < 0.05; **P < 0.01; ***P < 0.001.

We successfully knocked down filaggrin with designed siRNA (**Figures S1, 2**) and then evaluated the effect for related molecules. Knock-down filaggrin not only suppressed the filaggrin expression (**Figure 7**) but also suppressed the expression of E-cadherin (**Figure 8**). Moreover, *filaggrin* deficiency further decreased the expression of E-cadherin in BEAS-2B cells with IL-4 inducing. Interestingly, *filaggrin* deficiency did not affect the expression of IL-33 and TSLP in BEAS-2B cells apparently (**Figure 8**). However, when the BEAS-2B cells were incubated with IL-4, the expression levels of IL-33 and TSLP were elevated notably by knock-down filaggrin with specific siRNA which suggested that filaggrin was probably involved in the regulation mechanism of IL-4 to IL-33 and TSLP.

## Disscusion

Asthma is a chronic inflammatory disease of the airways that affects more than 300 million people worldwide (29). It has been known that airway eosinophilia is associated with an increasing risk of asthma exacerbations (30). As the previous studies, asthma can be divided into eosinophilic asthma (EA), neutrophilic asthma (NA), paucigranulocytic asthma (PGA) and mixed-granulocytic asthma (MGA) based on the relative cell counts of induced sputum (1). Among the four inflammatory phenotypes, EA is found in 17-36% of asthma patients who have severe airflow obstruction despite optimal treatment and more frequent exacerbations (31). In our previous research, 36.9% asthmatic patients were defined as EA in 176 asthma subjects (1). It is known that the eosinophilic airway inflammation is highly correlated with childhood-onset and severe asthma, so we focused on this specific group and explored the pathogenic mechanisms of EA.

Since asthma is well-known as a heterogeneous polygenic disease, plenty of genetic studies of asthma in different populations have documented an association with polymorphisms of *IL4, IL13, ADRB2, TNF, HLA-DRB1, FCER1B, L4RA, CD14, HLA-DQB1* and *ADAM33* genes (2). These genes implicate abnormalities in epithelial barrier function and innate and adaptive immune responses which reveal the pathogenic mechanism of asthma. Although a series of genetic loci have been identified with involvement of asthma in different population groups, so far, the genomic background of EA has not been clarified yet. To analyze the genomic variations of EA patients, and their correlation with clinical characteristics, in this study targeted next-generation sequencing (including the promoter and exon regions of 109 asthma candidate genes) was used to detect the peripheral blood of 77 Chinese EA patients, and compared with 431 Chinese healthy controls to obtain differential genomic variations. We identified differential genomic variations in EA patients included 63 single nucleotide variants (SNVs) and 14 Insertion-Deletions (InDels) (**Table S2**) affecting protein translation. There were 41 differential SNPs screened with mutant patients more than 3. And among these screened SNPs, *FLG* rs192116923-G allele was found for the first time to be associated with EA patients with AD. On the other hand, *FLG* rs75235053-G allele and *GSTM1* rs412302-A allele were associated with severe EA. These finding suggested that filaggrin was implicated in the pathogenesis of EA.

Filaggrin is a crucial epidermal protein encoded by *FLG* gene that is important for the formation of the corneocyte, as well as the generation of its intracellular metabolites, which contribute to stratum corneum hydration and pH (32). Loss-of-function mutations in 1 or both alleles of *FLG* result in reduced or completely absent levels of epidermal filaggrin, respectively. Recent GWAS reports found *FLG* loss-of-function mutations were closely related to childhood-onset and severity of asthma (33). *FLG* R501X and 2282del4 account for about 48% of AD patients and 9% of European and American Caucasians, but were hardly seen in Asian and African people (34, 35). Since the genetic association of SNPs in *FLG* gene and asthma has been concerned in previous researches, we analyzed the biological function of *FLG* rs75235053 C>G and rs192116923 T>G by bioinformatics tools. Interestingly, *FLG* rs75235053 C>G appeared to be a new splicing site, suggesting that it may cause mRNA truncation and reduce filaggrin monomers, thereby affecting the epidermal barrier function. *FLG* rs192116923 T >G may increase the binding to Smad3 or Smad2_Smad3_Smad4 complex. Smad3 is a nuclear transcription factor necessary for TGF-β1 signaling and can be triggered by TGF-β1. Activated and phosphorylated Smad3, Smad2 and Smad4 form a trimeric complex, which can enter the nucleus and induce gene transcription after binding to DNA under the action of DNA-binding cofactors (36). Recent evidence indicated that TGF-β1 could decrease filaggrin protein expression in the late stage of epidermal differentiation (37), so the combination of Smad3 may have an inhibitory effect on filaggrin expression.

Furthermore, it was found that *FLG* gene deficiency can affect epidermal cell migration, adhesion and proliferation, promote apoptosis, and interfere with the cell cycle (37). Considering the critical roles of filaggrin in epithelial cells, whether the respiratory epithelial cells expressed filaggrin and the further regulatory mechanisms still needs further exploration. Although some studies proposed that filaggrin expression was absent in the upper airway epithelial cells in both healthy controls and asthma patients (38), in our study it showed opposite results. In this article, it was firstly reported that human airway epithelial cell line BEAS-2B cells constitutively expressed filaggrin, and its expression is not less than human immortalized epidermal cell line HaCaT cells. More importantly, we have proved that filaggrin is also highly expressed in human normal bronchial epithelial cells *in vivo*. This finding created the basis for further investigation of filaggrin in asthma.

On the other hand, asthma is generally divided into T helper type 2(Th2/T2)-high and T2-low endotypes according to the cytokines involved in the pathogenesis of asthma (39). EA patients usually exhibit the secretion of typical Th2 cytokines, such as IL-4, IL-5 and IL-13, and induce Th2 immune response, which then generates a series of cascades downstream, including IgE-stimulated hypersensitivity, airway epithelial cell activation, effector cell (including mast cells, eosinophils and basophils) chemotaxis and epithelial and subepithelial matrix remodeling (10). It has been proved that AD skin is characterized by the over-expression of IL-4 and IL-13 while these Th2 cytokines significantly reduced filaggrin gene expression in keratinocytes which indicated that filaggrin was downregulated by Th2 milieu (14). Since Th2 cytokines also play vital roles in the pathogenesis of asthma, to exploring how filaggrin expression was regulated in the bronchial epithelial microenvironment by Th2 cytokines and whether the influenced filaggrin level elicited any downstream reaction to maintain the inflammatory response in asthma, we mimicked the Th2 cytokine environment with IL-4 and demonstrated that IL-4 suppressed the expression of filaggrin in bronchial epithelial cell line BEAS-2B cells.

In this study, it was found that IL-4 not only suppressed filaggrin expression but also disrupted the expression of E-cadherin. E-cadherin is the main adhesion junction protein of airway epithelium and an important part of the airway epithelial connection complex (40). E-cadherin establishes the connection between cells, promotes cell polarization, and regulates the transport of macromolecules and ions around cells, thereby maintains the normal function of airway epithelial barrier. Early epithelial-mesenchymal transition (EMT) is related to the loss of E-cadherin, and the reduction of E-cadherin can promote the transformation of M2-type macrophage (41, 42). They all play an important role in the pathogenesis of asthma.

Consistent with previous studies *in vivo* in AD (43), we found that IL-4 remarkably upregulated IL-33 and TSLP in BEAS-2B cells. Researchers also found that IL-4 increased the expression of IL-33 in keratinocytes and they conjected that there may be a positive feedback loop connected IL-4 and IL-33 in AD (44, 45). It has been known that the epithelial cytokines IL-33 and TSLP are warning factors for external stimuli and key regulators of Th2 inflammatory response, and play a role in tissue repair after injury (46). In the Th-2 environment in AD, epithelial IL-25, IL-33, and TSLP are upregulated. IL-33 can activate group 2 innate lymphoid cells (ILC2s) to produce Th2-type cytokines, such as IL-5, IL-13, and up-regulate the expression of OX40 and PD-L1 on the cell surface (47, 48). On the other hand, in the presence of IL-33, antigens can activate Th2-polarized CD4+T cells to participate in Th2 immune responses (49). Moreover, it has been reported that both IL-33 in human keratinocytes can downregulate the expression of filaggrin through phosphorylation of STAT3 and ERK (50). TSLP is also an endogenous mediator in patients with AD and a master regulator of Th2-driven inflammation (43). More importantly, TSLP is a direct trigger factor of T cell migration in *FLG*-deficient skin analogs (51).

However, the role of filaggrin in the regulation of IL-4 to E-cadherin and the epithelial-derived cytokines is still indistinct. Thus, we designed the specific filaggrin siRNA and successfully knocked down filaggrin expression in BEAS-2B cells. Then it was proved that the deficiency of filaggrin only effected E-cadherin but did not significantly impact IL-33 or TSLP. Moreover, E-cadherin was more observably reduced by filaggrin deficiency when induced with IL-4. And simultaneously, the expressions of IL-33 and TSLP were more significantly enhanced in effect of IL-4 when filaggrin expression was suppressed. These findings raised the possibility that the destruction of airway epithelial barrier and the promotion of Th2 immune response caused by *FLG* mutations was also part of the mechanism of asthma.

The molecular mechanism of IL-4 regulating filaggrin and other molecules was not clear yet and we are still working on this issue. We have proved that the filaggrin siRNA did not change the IL-4R expression in BEAS-2B cells. Therefore, filaggrin knockdown did not affect the responses of BEAS-2B cells to IL-4 incubation *via* regulation of IL-4R. Previous studies have shown that increased apoptosis of bronchial epithelial cells also played important roles in asthma pathogenesis. Recently, Jiapeng Hu and his colleagues proved that IL-4 or IL-13 stimulation induced the overexpression of fructose-1,6- bisphosphatase (Fbp1) which aggravated the oxidative stress and apoptosis in bronchial epithelial cells (52). In our study, we aslo found that IL-4 treatment and filaggrin siRNA transfection suppressed the viability of BEAS-2B cells. We speculated that knock-down of filaggrin may also lead to the oxidative stress induced apoptosis as the same as IL-4 treatment, which still needs further exploration.

Moreover, whether this positive feedback mechanism occurred in normal human bronchial cells is still unclear. We tried to perform these experiments in primary normal human bronchial epithelial (NHBE) cells, but the isolated bronchial epithelial cells were probably contaminated with fibroblasts and other stroma cells and were hard to identify. Further explorations are needed to answer this question.

**Figure 9:**
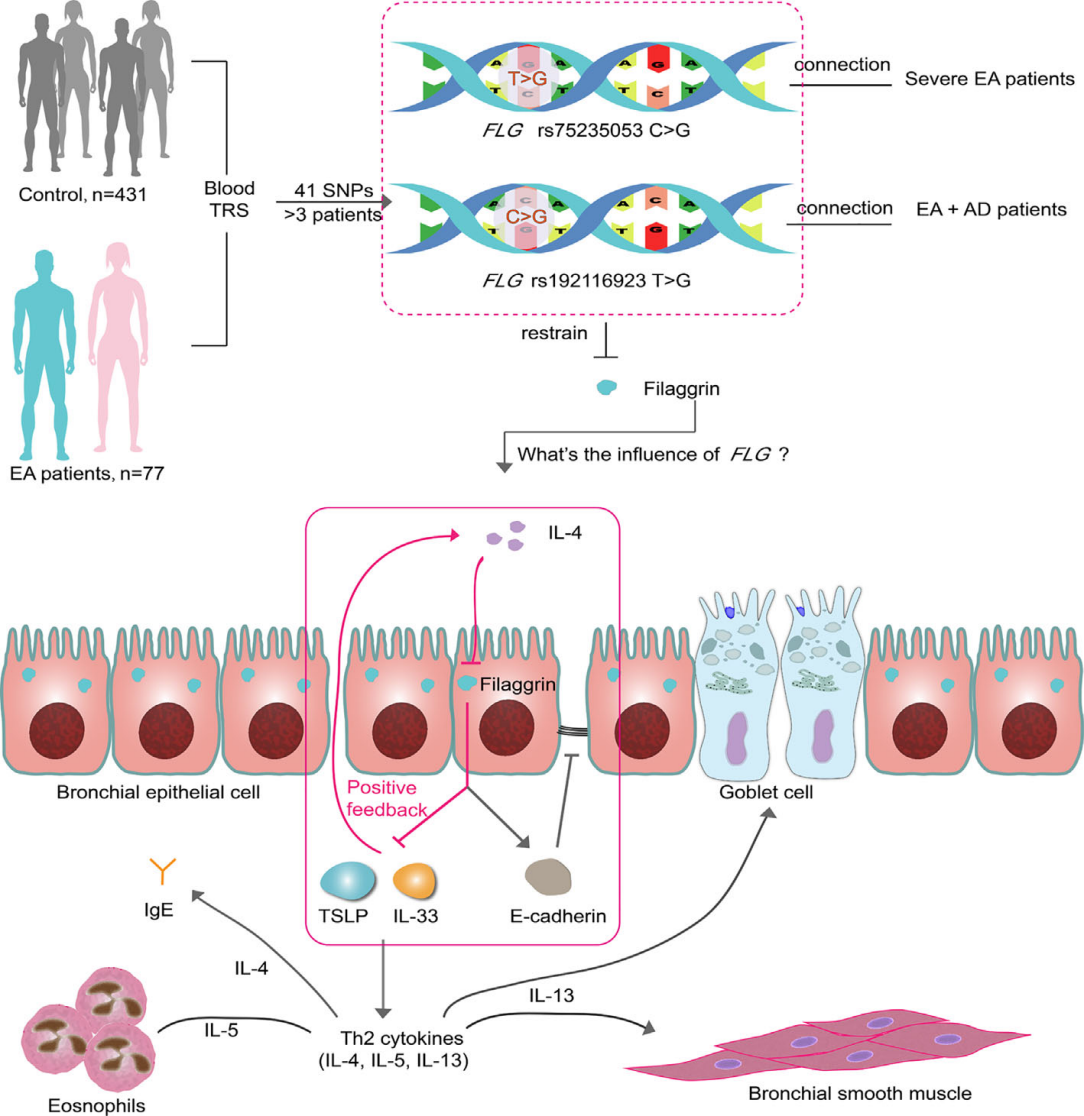
Two SNPs may influence EA patients *via* filaggrin through a positive feedback mechanism. There are 41 SNPs indentified from 77 EA patients by TRS. Two of them in *FLG* gene are associated with the clinical characteristics of EA patients. In this article, it has been proven that filaggrin expression is inhibited by the Th2 cytokine IL-4 in human bronchial epithelial cells, while the reductive filaggrin increases the expression of epithelial derived cytokines TSLP and IL-33 which induce the releasing of Th2 cytokines such as IL-4 and form a positive feedback loop (red box). The declining filaggrin also intensifies the reduction of E-cadherin induced by IL-4. EA, Eosinophilic asthma; AD, atopic dermatitis; TRS, target region sequencing.

In conclusion, we proposed that human normal airway epithelial cells *in vitro* and *in vivo* constitutively expressed filaggrin. The Th2 cytokines, IL-4, could reduce the expression of filaggrin and E-cadherin and induce the expression of epithelial-derived T2 cytokines in airway epithelial cells. Furthermore, filaggrin deficiency enhanced Th2 inflammatory response *via* upregulating the expression of IL-33 and TSLP in the presence of Th2 cytokines. After all, we conjected that the loss of function mutations of *FLG* gene we found in previous study in EA patients may destruct the integrity of airway epithelia not only *via* deficiency of filaggrin but also *via* suppression of E-cadherin. Furthermore, filaggrin deficiency could intensify the Th2 responses by induction of inflammatory molecules IL-33 and TSLP under the Th2 condition, which forms a positive feedback regulatory loop to amplify the immune response and promotes the progression of asthma (**Figure 9**).

## Data Availability Statement

The datasets presented in this study can be found in online repositories. The names of the repository/repositories and accession number(s) can be found below: BioProject accession PRJNA718105.

## Ethics Statement

The studies involving human participants were reviewed and approved by the Human Research Ethics Committee of the Chinese PLA general hospital. The patients/participants provided their written informed consent to participate in this study. Written informed consent was obtained from the individual(s) for the publication of any potentially identifiable images or data included in this article.

## Author Contributions

WG, JG, HH and PB contributed to conception and design of the study. WG performed the major parts of the experiments. JG organized the database. WG and JG performed the statistical analysis. PB contributed to critical parts of the study implementation. WG wrote the first draft of the manuscript. JG, MM, YZ, and PB wrote sections of the manuscript. All authors contributed to manuscript revision, read, and approved the submitted version.

## Funding

This study was supported by Logistic Support Department of CMC Health Care Project (No.21BJZ42), National Natural Science Foundation of Beijing (No.7212104) and the Special Program for Innovation of the National Defence Science and Technology of China(No.20-163-12-ZD-029-006-01).

## Conflict of Interest

The authors declare that the research was conducted in the absence of any commercial or financial relationships that could be construed as a potential conflict of interest.

## Publisher’s Note

All claims expressed in this article are solely those of the authors and do not necessarily represent those of their affiliated organizations, or those of the publisher, the editors and the reviewers. Any product that may be evaluated in this article, or claim that may be made by its manufacturer, is not guaranteed or endorsed by the publisher.
