# Supplementary material for: The Pathogenesis of Eosinophilic Asthma: A Positive Feedback Mechanism That Promotes Th2 Immune Response via Filaggrin Deficiency
Source: Front Immunol. 2021 Aug 13;12:672312. doi: 10.3389/fimmu.2021.672312 (PMC8414997; doi:10.3389/fimmu.2021.672312)
Supplement: Supplementary file 1 [file DataSheet_1.docx]

**Table S1. Asthma candidate genes (109 genes involved in asthma according to previous reports including several large-scale GWAS studies)**

| *ORMDL3 TLR4 CCL4 IL12RB2 CYP11A1 TLR2 CCL7 TSC1 CCR3 TSLP CCL17 PTGDR2 CCR4 VDR CCL22 RORC ADAM33 CDHR3 CCL24 TNFSF10 GATA3 CREB1 CXCL10 TBX21 GATA6 CTLA4 IL5RA GPRA STAT6 CXCR2 IL25 GPR65 ADAM8 CYP17A1 IL33 FCERIB CCL5 DUSP1 IL37 DPP4 CCL11 GSDMB CRTH2 CLCA1 IL12A GSTM1 PGD2 SERPINB2 IL12B HSD3B1 C3 HAVCR2 IL13 FOXP3 CXCL1 IFNG IL17A IL10 CXCL8 IRAKM IL1RL1 IL6 MMP9 IRF2 IL4 CHI3L1 IL18 PHF11 IL5 STAT4 LTE4 ATG5 IL21 FLG IL4RA ATG7 IL23 IL2 MTORC1 SPINK5 POMC NFKB1 TLR9 ZNF365 TGFB1 CCL2 IL12RB1 ZPBP2 IFI16 SYK TRPV1 TRPV2 ICAM1 SCGB3A2 CASP4 ADA PLA2G7 DPP10 NLRP3 MMP1 BDNF HLA-G MPO PF4 VCAM1 FAS ITK MS4A2 SMC3* |
| --- |

**Table S2. Differential InDels of EA patients**

| Gene | av SNP | Function | REF/ALT | Alt N. | Ref rate | OR | P_value |
| --- | --- | --- | --- | --- | --- | --- | --- |
| *ADAM33* | rs146576636 | frameshift | ATCTGGACT/A | 19 | 0.120 | 11.588 | <0.001 |
| *HLA-G* | rs41557518 | frameshift | AC/A | 12 | 0.019 | 5.124 | <0.001 |
| *DPP4* | rs34282135 | splicing | G/GTT | 27 | 0.177 | 1.801 | 0.003 |
| *DPP4* | rs71408195 | splicing | G/GT | 8 | 0.003 | 18.21 | <0.001 |
| *ADAM8* | . | splicing | A/AGAG | 2 | - | Inf | <0.001 |
| *ADAM8* | . | splicing | A/AGAC | 2 | 0.068 | 0.18 | 0.013 |
| *IRF2* | rs750152068 | splicing | G/GAAA | 3 | 0.008 | 5.027 | 0.004 |
| *HAVCR2* | rs70984443 | splicing | A/AAG | 3 | 0 | Inf | <0.001 |
| *CDHR3* | rs201638418 | frameshift | GC/G | 2 | 0.004 | 6.64 | 0.011 |
| *IL5RA* | . | splicing | A/ACAC | 1 | - | Inf | 0.018 |
| *IL5RA* | . | splicing | A/AACT | 1 | - | Inf | 0.018 |
| *IL5RA* | . | splicing | T/TAAA | 3 | 0.002 | 9.916 | 0.014 |
| *IL5RA* | . | splicing | A/AACC | 3 | 0.006 | 6.716 | 0.002 |
| *IL5RA* | . | splicing | A/AACC | 1 | - | Inf | 0.018 |

av SNP: dbSNP ID.

Figure S1


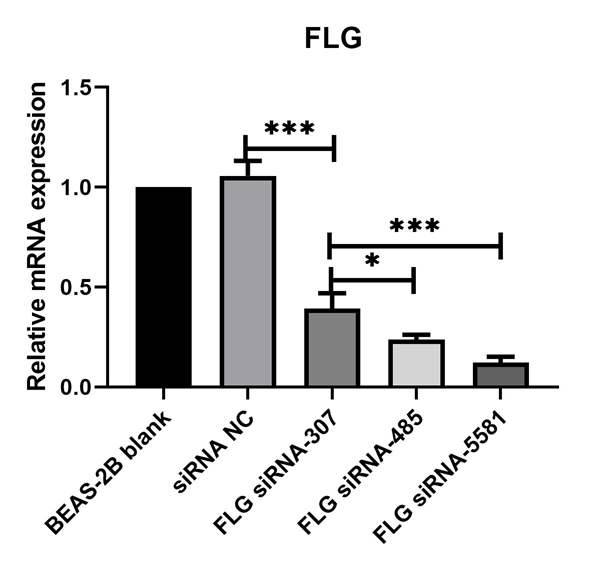


**Figure S1.Filaggrin mRNA expression after transfection of Filaggrin siRNA into BEAS-2B cells.**

BEAS-2B blank means a blank control group, siRNA NC means a negative group.

* P <0.05, *** P <0.001.

Figure S2





Figure S2. The protein expression of filaggrin was suppressed by specific designed siRNA. Western blot experiment has been repeated for 3 times and presented as the mean ± SD (n=3). The Student’s t-test was used to analyze the difference between groups. *P<0.05 was considered as statistically significant.

Figure S3


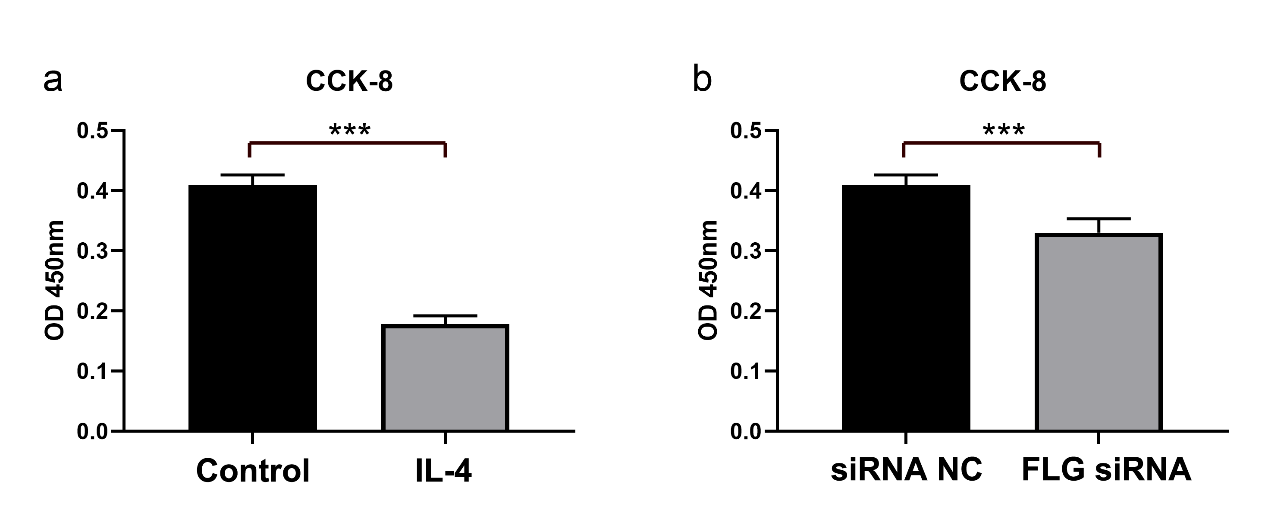
 **Figure S3. BEAS-2B cell viability decreased in IL-4 induced (a) and filaggrin knock-down groups (b).** The BEAS-2B cell viability in different groups were detected by CCK8 assay. *** P <0.001.

Cell Counting Kit-8 (CCK-8, Beyotime) was used for the detection of cell viability. BEAS-2B cells were seeded in 5 × 10^3^/wells of 96-well plates and preincubated for 24 h in a humidified incubator with 5% CO2 at 37 °C before the CCK-8 solution (10 μl) was added to each well of the plate. Then, the cells were incubated for 1 h, and the absorbance was measured at 450 nm with a microplate reader (BioTek Epoch, USA).

Figure S4


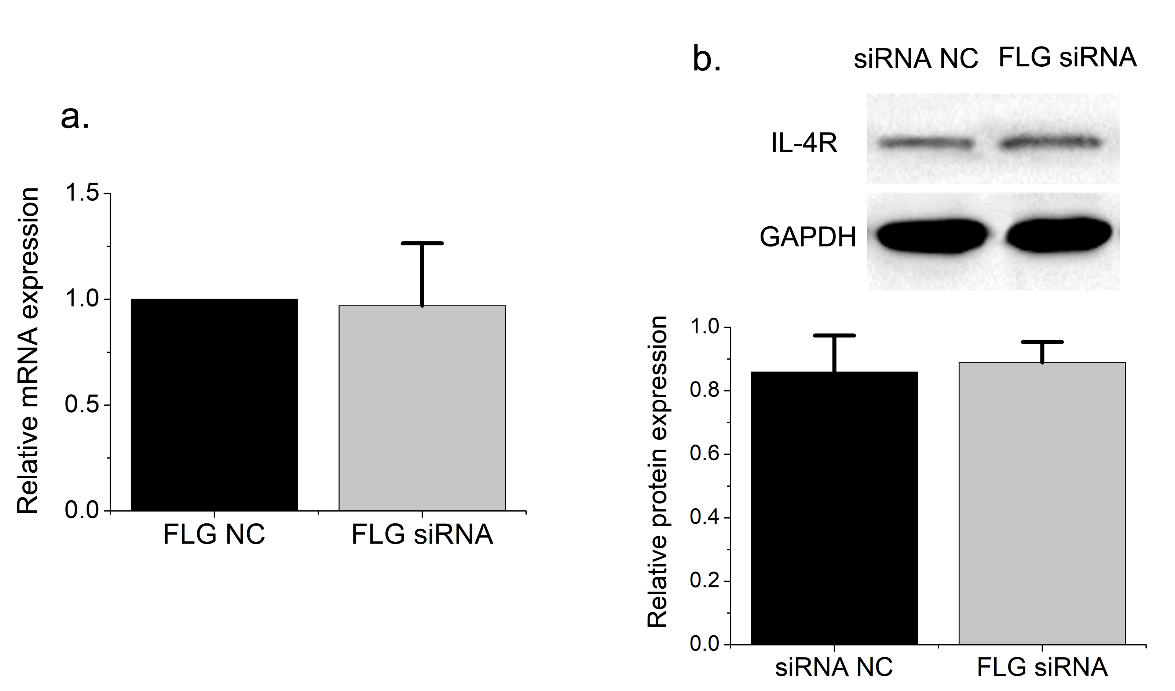


Figure S4. The expression of IL-4R was not been affected by filaggrin siRNA. The Student’s t-test was used to analyze the difference between groups. The mRNA expression of IL-4R was detected with RT-PCR and the protein expression of filaggrin was detected with Western blot. Each experiment has been repeated for 3 times and presented as the mean ± SD (n=3). There was no statistically difference between the two groups transfected with non-specific control and filaggrin siRNA.
